# Numerical evaluation of sweeping gas membrane distillation for desalination of water towards water sustainability and environmental protection

**DOI:** 10.1038/s41598-024-54061-5

**Published:** 2024-02-22

**Authors:** Yan Cao, Ali Taghvaie Nakhjiri, Mahdi Ghadiri

**Affiliations:** 1 School of Computer Science and Engineering, Xi’an Technological University, Xi’an, 710021 China; 2 Department of Petroleum and Chemical Engineering, Science and Research Branch, Islamic Azad University, Tehran, Iran; 3 Institute of Research and Development, Duy Tan University, Da Nang, 550000 Vietnam; 4 The Faculty of Environment and Chemical Engineering, Duy Tan University, Da Nang, 550000 Vietnam

**Keywords:** Heat transfer, Mass transfer, Mathematical modelling, Membrane distillation, Temperature, Chemical engineering, Computational science, Environmental chemistry

## Abstract

Sweeping gas membrane distillation (SGMD) is considered a membrane distillation configuration. It uses an air stream to collect the water vapour. A 2D mathematical model is prepared in the current study to predict the effect of various operating parameters on the SGMD performance. Also, the temperature distribution in the SGMD was obtained. The effect of air inlet temperature, salt concentration, feed and air flowrate on air and salted solution outlet temperature and vapour flux through the membrane is investigated. There was good agreement between experimental data and modelling outputs. It was found that increase in air inlet temperature from 40 to 72 °C was increased the outlet temperature of air stream and cold solution from 37 to 63 °C and 38 to 65 °C respectively. Furthermore, increase in air inlet temperature led to the enhancement of vapour flux in the membrane distillation. Also, the salt concentration and feed flow rate did not have meaningful influence on the outlet temperatures, however, the flux was increased by increasing feed flowrate.

## Introduction

Membrane distillation (MD) is considered one of the effective and alternative techniques for the treatment of water and wastewater containing high amount of dissolved salt. Traditional water desalination methods like reverse osmosis are too expensive and considerable membrane fouling can happen as it is a pressure driven system^1,2^. Driving force of MD separation process is vapour pressure differential between cold and hot sides and it is created due to the temperature difference involved in the process^3^. MD has been divided into different types based on the creation of pressure vapour difference of two side of the contactor^4–6^. The configuration of direct contact membrane distillation (DCMD) is called when a hot liquid on the feed side has direct contact with a cold solution on the permeate side of the contactor. There is a microporous hydrophobic membrane between feed and cold sides. Transmembrane flux is carried out because of the difference in temperature between the hot and cold streams which results in a vapour pressure difference between two sides of the microporous membrane^7^. When the vapour phase passed through membrane is vacuumed on the permeate side, it is called vacuum membrane distillation (VDM)^8^. In air/water gap MD (AGMD or WGMD), There are a microporous membrane as well as a condensation surface which air or water is confined between the membrane and condensation plate^9^. In terms of SGMD, an inert gas carrying permeated vapour into permeate side, and then, a condenser is used for converting to vapour into water^10^.

Sweep gas MD like direct DCMD uses both Knudsen and molecular diffusion for the transmission of vapour through the hydrophobic microporous membrane^11^. It was reported that SGMD has faster mass transfer rates than VMD while conduction heat loss is lower in SGMD than DCMD^12^. But, the heat as well as mass transfer resistances in the sweep gas can also have a considerable influence on the overall heat and mass transfer rates. It should be noted one of the benefits of SGMD is its relatively smaller conductive heat loss^13^. Therefore, investigation of the vapour heat and mass transfer is highly important to understand the process in details and improve the system performance. Firstly, this configuration was used in 1963 to produce drinkable water with an undrinkable aqueous solution. A tubular silicon was used as membrane and air as sweeping gas^14^.

Numerical solution and mathematical modelling are a useful and cost-effective method to gain further insights on the influences of the operating conditions and the parameters needed for the system design to enhance the performance of different membrane-based technologies like gas separation, liquid–liquid extraction and sweep gas membrane distillation^15,16^. Many modelling and simulation studies have been conducted to evaluate mass as well as heat transfer in DCMD^7,17,18^ and VMD^19^, however, there are little studies in terms of SGMD^20^.

Computational fluid dynamic (CFD) is a method used widely for the modelling and simulation of different configurations of membrane distillation. Baghel et al.^21^ used 3D CFD model for investigation of VMD. In the developed model, the theoretical permeate flux through membrane as well as interfacial temperatures were calculated by the constructed CFD model. Also, various operating factors were evaluated in terms of permeate flux as well as specific energy consumption. Ghadiri et al.^17^ constructed a numerical method for the prediction of the vapour penetration across membrane, concentration, temperature, and velocity distribution in the DCMD. Response surface methodology (RSM) was used to investigate and optimize a VMD system^11^. A 2D-tailored in-house CFD code was used for simulation of DCMD at unsteady condition in order to investigate heat and mass transport in plate-and-frame contactor. The impact of spacers on the fluid flow, polarization, and permeate production was evaluated and it was found that the spacers can increase permeated vapour through the membrane^22^. Polarization phenomena in DCMD were studied by the CFD technique^23^. The concentration polarization is an important parameter in the membrane distillation process because it decreases system efficiency and results in mineral scaling^23^. Fibre arrangement including row space and intersection angle effect on the temperature, concentration, and vapour polarization was investigated in VMD^24^. It was observed that fibre arrangement can have a significant influence on the module performance^24^. Lee et al.^25^ suggested a one-dimensional steady-state simulation model to study multi-stage air gap membrane distillation. As there are few studies on the investigation of SGMD in the literature in terms of modelling and simulation, it will be useful to develop a CFD model for the evaluation of operating parameters’ effect on the process performance.

The current work presents comprehensive numerical modelling and simulation for the investigation of operating variables like inlet temperature of feed solution, salt concentration, and velocity of liquid and gas streams on the SGMD system performance. Polyvinylidene fluoride (PVDF) has been used as the microporous membrane in the system^20^. Temperature and velocity distribution were obtained at various operating conditions.

## Model development

**Figure 1:**
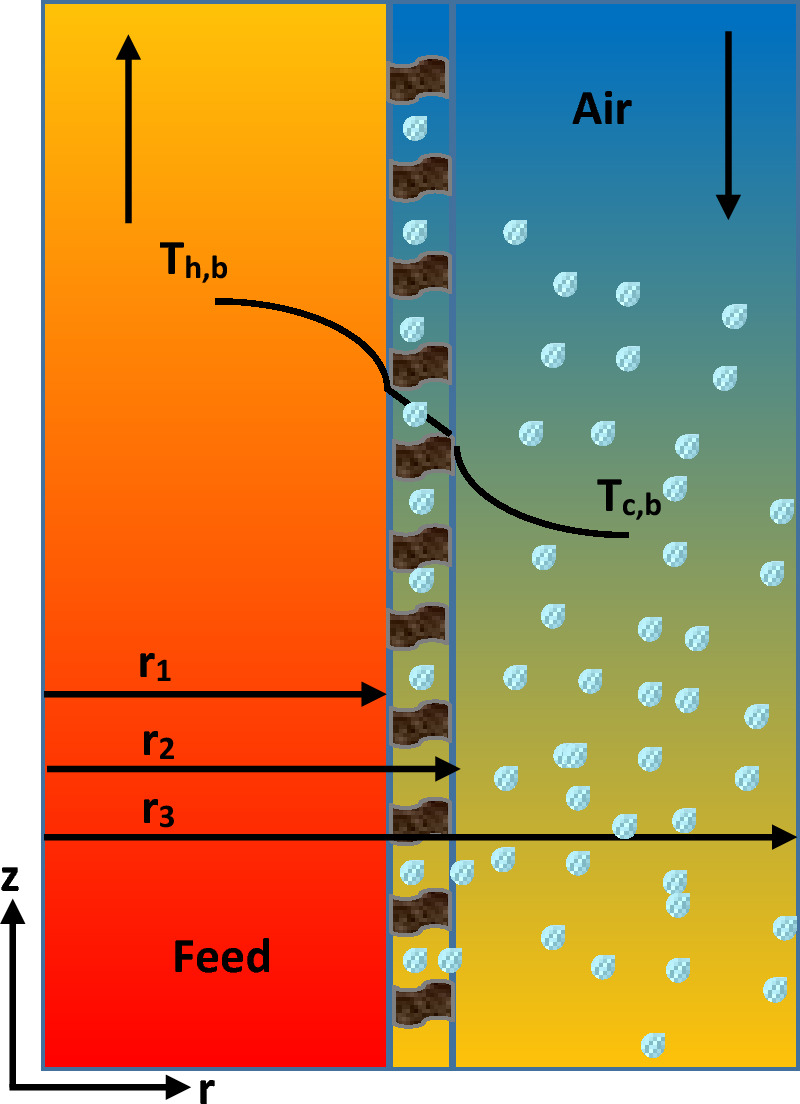
Sweeping gas membrane distillation configuration.

A two-dimensional mathematical model was constructed to evaluate transport phenomena in the SGMD system. Convection, conduction, and diffusion terms were considered for the assessment of heat and mass transfer in combination with momentum equation. As momentum equation is required for determination of temperature as well as concentration distribution in the MD. The derived transport equations were solved in three subdomains including aqueous solution side (tube side), membrane, and air stream side (shell side) where air flows. The air fills microporous membrane due to its hydrophobic property. Figure 1 presents the overall schematic of sweeping gas membrane distillation. The saline water passes across tube side while the air stream enters the shell side of membrane distillation at counter-current mode. There is a contact between liquid and air on the membrane surface. The evaporation happens due to the temperature difference between two phases. The produced vapour penetrates through membrane pores and enter into the shell side, then, air stream carries the permeated to remove from the contactor. The evaporation phenomenon decreases the temperature of feed across the membrane. Table 1 provides operating conditions, membrane specification, and the fluid properties which used in the developed model.

**Table 1:** Physical properties, operating conditions and membrane specification.

Parameter	Unit	Value
Thermal conductivity of PVDF	W/m K	0.180
Thermal conductivity of water vapour	W/m K	0.027
Membrane material	–	PVDF
Module inlet diameter	m	0.01
Fibre length	m	0.127
Fibre inner diameter	m	2.3e−4
Fibre outer diameter	m	3.3e−4
Porosity (%)	–	55
Number of fibres	–	10,633
Feed inlet temperature	°C	40–72
Air inlet temperature	°C	24
Feed inlet velocity	m/s	0.13–0.78
Air inlet velocity	m/s	0.008 and 0.02
NaCl concentration	g/L	0–140

### Feed side equations

The heat and mass transfer equations were derived for the feed side as follows^26,27^:

1 $$\frac{1}{r}\frac{\partial }{\partial r}\left({k}_{f}r\frac{\partial T}{\partial r}\right)+\frac{\partial }{\partial z}\left({k}_{f}\frac{\partial T}{\partial z}\right)={\rho }_{f}{C}_{p, f}{V}_{z,f}\frac{\partial T}{\partial z}$$

2 $$\frac{1}{r}\frac{\partial }{\partial r}\left({D}_{f}r\frac{\partial {C}_{A,f}}{\partial r}\right)+\frac{\partial }{\partial z}\left({D}_{f}\frac{\partial {C}_{A,f}}{\partial z}\right)={V}_{z,f}\frac{\partial {C}_{A,f}}{\partial z}$$

where ρ_*f*_ (kg m^−3^), C_*p,f*_ (kJ kg^−1^ K^−1^), V_*z,f*_ (m s^−1^), *k*_*f*_ (W m^−1^ K^−1^), D_f_ (m^2^/s), T (K), and C_A,f_ are density, the heat capacity, flow velocity vector along membrane contactor, the thermal conductivity coefficient, the water diffusion coefficient in the feed, temperature, and water concentration in the aqueous solution.

The velocity of feed solution (V_z,f_) in the lumen side along membrane contactor (Eq. 3) is determined by parabolic laminar flow^28–30^:

3 $${V}_{Z,f}=2u\left[1-{\left(\frac{r}{{r}_{1}}\right)}^{2}\right]$$

where *u* (m/s) denotes average velocity of feed solution, and *r*_*1*_ (m) refers the inner radius of lumen.

The diffusion coefficient (cm^2^/s) was estimated using Wilke–Chang method based on the following equation^28^:

4 $$D_{water - i} = \frac{{7.4 \times 10^{ - 8} \left( {\emptyset M_{i} } \right)^{0.5} T}}{{\eta_{i} V_{water}^{0.6} }}$$

where *i* can be feed or permeate side of membrane.

### Membrane equations

The membrane was a microporous media and the conductivity was considered as only mechanisms for the heat transfer. In addition, mass transfer only happens using diffusion mechanism. Therefore, the heat and mass transfer equations can be written as follows for the membrane side^26,31^:

5 $$\frac{1}{r}\frac{\partial }{\partial r}\left({k}_{m}r\frac{\partial T}{\partial r}\right)+\frac{\partial }{\partial z}\left({k}_{m}\frac{\partial T}{\partial z}\right)=0$$

6 $$\frac{1}{r}\frac{\partial }{\partial r}\left({D}_{m}r\frac{\partial {C}_{A,m}}{\partial r}\right)+\frac{\partial }{\partial z}\left({D}_{m}\frac{\partial {C}_{A,m}}{\partial z}\right)=0$$

where *k*_*m*_ (W/m K) denotes the thermal conductivity coefficient, D_m_ (m^2^/s) is the water diffusion. The thermal conductivity coefficient is determined as follows^26,31^:

7 $${\text{k}}_{{\text{m}}} = \left( {\left( {{1} - \varepsilon } \right) \times {\text{k}}_{{\text{s}}} } \right) + \left( {\varepsilon \times {\text{k}}_{{\text{g}}} } \right)$$

where k_g_ and k_s_ refer the thermal conductivity coefficient of vapour and PVDF respectively.

### Permeate side equations

The heat and mass transfer equations were derived for the gas stream on the permeate side as follows^26,31^:

8 $$\frac{1}{r}\frac{\partial }{\partial r}\left({k}_{p}r\frac{\partial T}{\partial r}\right)+\frac{\partial }{\partial z}\left({k}_{p}\frac{\partial T}{\partial z}\right)={\rho }_{p}{C}_{p, p}{V}_{z,p}\frac{\partial T}{\partial z}$$

9 $$\frac{1}{r}\frac{\partial }{\partial r}\left({D}_{p}r\frac{\partial {C}_{A,p}}{\partial r}\right)+\frac{\partial }{\partial z}\left({D}_{p}\frac{\partial {C}_{A,p}}{\partial z}\right)={V}_{z,p}\frac{\partial {C}_{A,p}}{\partial z}$$

In order to find gas stream velocity, Navier–Stokes equations were used as follows^28^:

10 $$\nabla .{V}_{Z,p}=0$$

11 $${\uprho }_{p} \frac{{\partial V_{Z,p} }}{\partial t} - \nabla .\left[ {\eta_{p} \left( {\nabla V_{Z,p} + \left( {\nabla V_{Z,p} } \right)^{T} } \right)} \right] + \rho \left( {V_{Z,p} .\nabla } \right)V_{Z,p} + \nabla p = F$$

where the symbols* η*_*p*_*, ρ*_*p*_*,* and* p* are to viscosity of solution (Pa s), density of solution (kg m^−3^), and pressure (Pa) of gas stream respectively.

### Boundary conditions

The boundary conditions of heat, mass, and momentum transfer for the feed, microporous membrane, and permeate side are provided in Table 2. The vapour flux was calculated using an equation reported by Esfandiari et al.^7^. The water vaporization enthalpy (H_v_) was determined as follows:

12 $${\text{H}}_{{\text{v}}} \left( {\text{T}} \right) = \left( {{1}.{7535} \times {\text{T}}} \right) + 2024.3$$

**Table 2:** Employed boundary conditions of heat, mass, and momentum transfer in different domains.

Position	Heat	Mass	Momentum
r = 0, z = 0 − L	Symmetrical	Symmetrical	–
r = 0 − r_1_, z = 0	T_f,in_	C_A,0_	–
r = 0 − r_1_, z = L	$\frac{\partial {T}_{h}}{\partial z}=0$	n.(− D_h_∇C_A,h_) = 0	–
r = r_1_, z = 0 − L	− J × H_v_	− J	Wall
r = r_1_ − r_2_, z = 0	Insulation	Insulation	Insulation
r = r_1_ − r_2_, z = L	Insulation	Insulation	Insulation
r = r_2_, z = 0 − L	J × H_v_	J	Wall
r = r_2_ − r_3_, z = 0	$\frac{\partial {T}_{p}}{\partial z}=0$	n.(− D_p_∇C_A,p_) = 0	Pressure, no viscous stress
r = r_2_ − r_3_, z = L	T_p,in_	0	V_p,in_
r = r_3_, z = 0 − L	Insulation	Insulation	Wall

### Numerical method

MATALB software was used to solve the constructed model equations in the aqueous solution, microporous membrane, and air stream sides with the defined boundary conditions. The MATLAB applied the central finite difference method to discretize the equations. The equations were simultaneously solved in three layers due to specific boundary conditions. In the numerical solution, 40 nodes were defined in r direction and there were 200 nodes along membrane contactor.

## Results and discussion

### Effect of saline water inlet temperature

**Figure 2:**
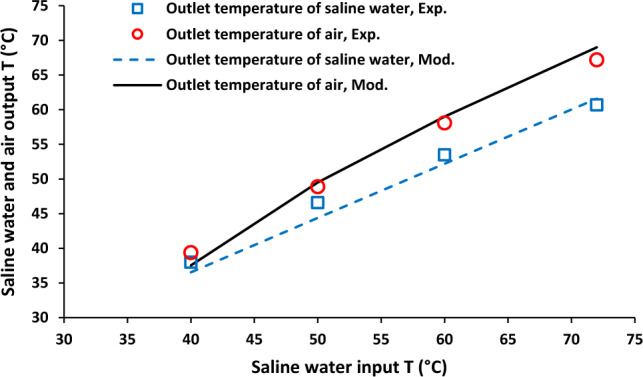
Saline water and air output temperature as a function of saline water inlet temperature, Experimental results reported in^20^.

**Figure 3:**
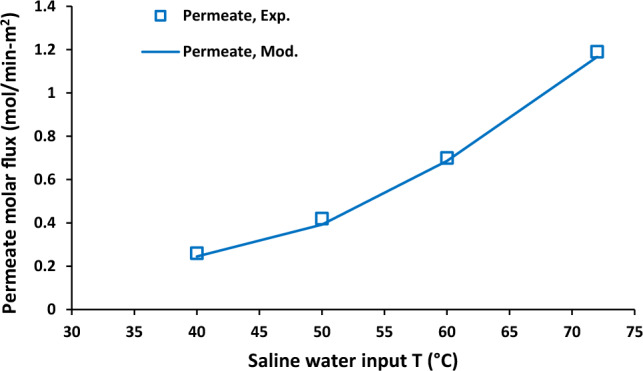
Permeate molar flux as a function of saline water inlet temperature, Experimental results reported in^20^.

Effect of saline water inlet temperature on outlet temperature of saline water and air stream was shown in Fig. 2. Brine solution inlet temperature was increased from 40 to 72 °C. The increasing of inlet temperature of brine solution led to the enhancement of both hot solution (from 36.53 to 61.59 °C) and cold side from 37.53 to 69.10 °C. Furthermore, it was observed that the difference between outlet temperatures of air stream and saline water was increased with the enhancement of saline water inlet temperature. Figure 3 shows permeate molar flux as a function of saline water inlet temperature. There was an increase in permeate molar flux from around 0.2–1.2 mol/min-m^2^ with increasing aqueous solution inlet temperature 40–72 °C.

**Figure 4:**
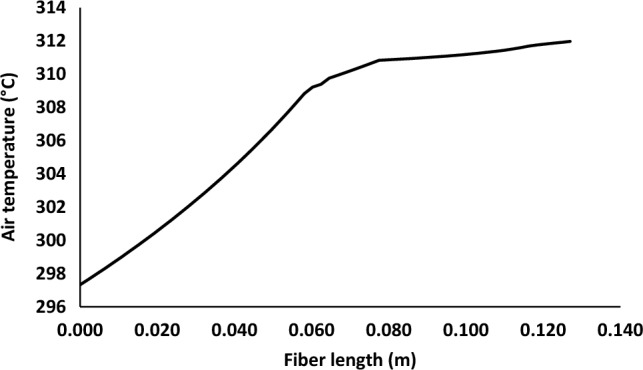
Air temperature profile along membrane contactor, solution inlet temperature = 40 °C.

Figure 4 shows temperature profile along membrane in the air side of membrane contactor. There is a sharp increase in temperature between entrance and 0.06 m. Then, it was observed gradual increase in temperature from around 309–312 K.

**Figure 5:**
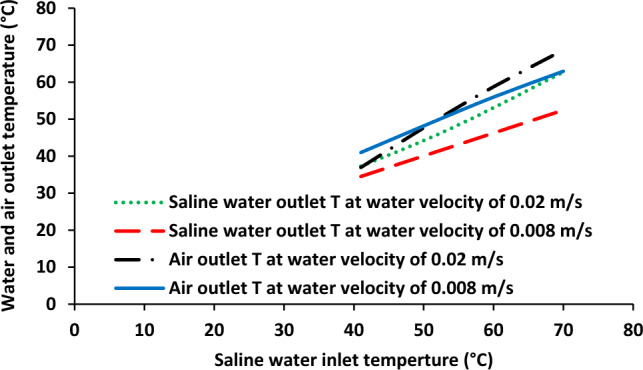
Saline water and air output temperature as a function of saline water inlet temperature at two different saline water flowrates.

**Figure 6:**
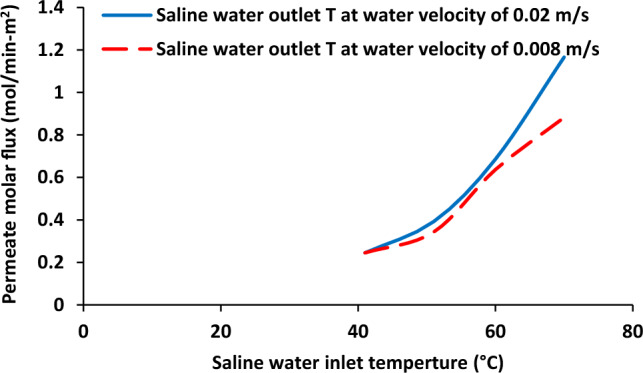
Permeate molar flux as a function of saline water inlet temperature at two different saline water flowrate.

Figures 5 and 6 show outlet temperature of both fluids and permeate flux as a function of saline water inlet temperature at two different water flowrates. As shown in Fig. 5, the outlet temperature is quite similar for both fluids at lower inlet temperature of saline water but the difference between saline water and air was increased with increasing of saline water inlet temperature at 0.02 m/s. When the saline water velocity was 0.008, the air outlet temperature was higher than saline water outlet temperature in the whole range of saline water inlet temperature. Also, the same behavior was observed in terms of difference in outlet temperatures of both fluids. Furthermore, based on the modeling results, it was found that the decrease in saline water solution can results in the reduction of water vapor permeation though microporous membrane pores.

### Effect of salt concentration

**Figure 7:**
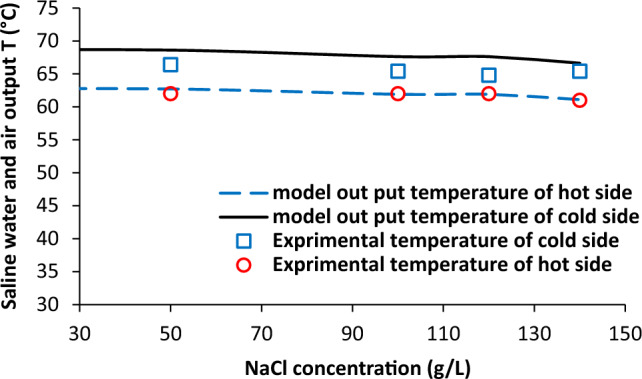
Saline water and air output temperature as a function of NaCl concentration, experimental results reported in^20^.

**Figure 8:**
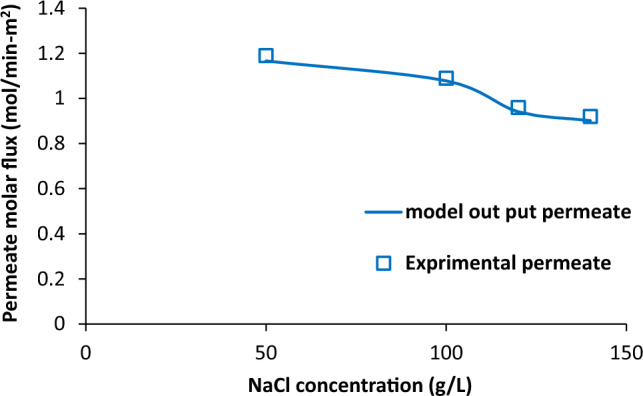
Permeate molar flux as a function of salt concentration, experimental results reported in^20^.

The dependence of saline water and air output temperature on NaCl concentration is given in Fig. 7. The salt concentration does not have any meaningful influence on the outlet temperatures in both side of membrane contactor. The variations of permeate molar flux with salt concentration is shown in Fig. 8. There was slight decrease in permeate molar flux (from 1.08 to 0.91 mol/min-m^2^) with increasing salt concentration from 50 to 140 g/L. It can be concluded that salt concentration in solution has negative impact on permeation on water vapours through membrane pores.

### Effect of saline water flowrate

**Figure 9:**
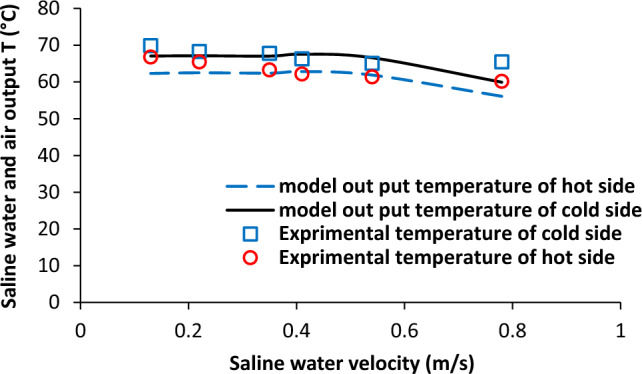
Influence of saline water velocity on saline water and air output temperature, experimental results reported in^20^.

**Figure 10:**
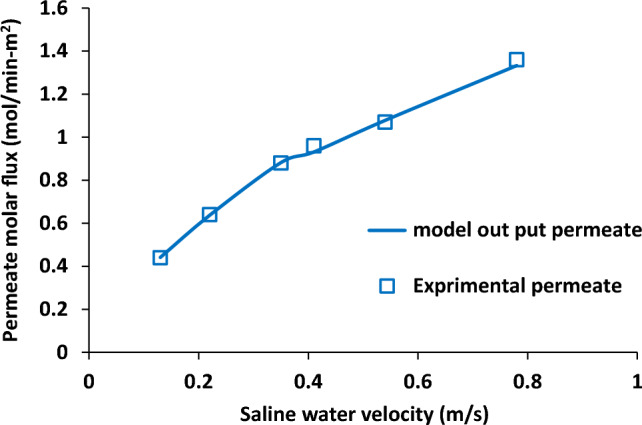
Influence of saline water velocity on permeate molar flux, experimental results reported in^20^.

Saline water velocity is one of the key flow parameters in membrane distillation system and it was analysed in this study. Figure 9 shows saline water velocity effects on temperature of aqueous solution and air stream. It was not observed change in outlet temperature of aqueous solution and air stream with the enhancement of saline water velocity from 0.13 to 0.54 m/s, but, there was slight decrease in both fluid between 0.54 and 0.78 m/s. Also, the difference between two fluids outlet temperature was quite same in the all range of saline water velocity. Furthermore, increase in saline water velocity led to the significant increase in the water vapour permeation flux from 0.4 to 1.4 mol/min.m^2^ as shown in Fig. 10.

## Conclusion

Desalination of seawater has been considered major priority for researchers due to the increasing demand for potable water. An in-depth model was established in the current work to assess effect of a number of operating parameters on outlet temperature of saline water and air stream in a SGMD. The obtained results demonstrated that the modelling outputs are quite similar with experimental results. The difference between outlet temperature of saline water and air was increased with increasing of aqueous solution inlet temperature. Also, it led to the enhancement of water vapour permeation through pores of membrane. The NaCl concentration in aqueous solution has a slight influence on outlet temperature of two fluid on both of membrane contactor but it was decreased water vapour permeation through the membrane from 1.078 to 0.892 mol/min m^2^. The water vapour permeation was increased considerably from 0.4481 to 1.333 mol/min m^2^ with the enhancement of saline water velocity from 0.13 to 0.78 m/s. Additionally, it was perceived from the results that increase in the velocity value of saline water from 0.15 to 0.8 m/s significantly improved the molar flux of permeate 0.4–1.4 mol/m^2^ min (Supplementary Information S1).

### Supplementary Information

Supplementary Information.

## Data Availability

The datasets used and analyzed during the current study are available from the corresponding author upon reasonable request.
