# Supplementary material for: Numerical evaluation of sweeping gas membrane distillation for desalination of water towards water sustainability and environmental protection
Source: Sci Rep. 2024 Feb 22;14:4340. doi: 10.1038/s41598-024-54061-5 (PMC10881985; doi:10.1038/s41598-024-54061-5)
Supplement: Supplementary file 1 — Supplementary Information. [file 41598_2024_54061_MOESM1_ESM.pdf]

Supplementary Material

Table S1. Values of independent and dependent variables

Independent Variables							Dependent Variables	
T_{bin} (˚C)	T_{bout} (˚C)	T_{ain} (˚C)	T_{aout} (˚C)	u_a (m/s)	u_b (m/s)	[NaCl] (g/L)	Permeate Molar Flux (mol/min-m^2)	Water Quality (mg/L)
72	60.7	24	67.2	0.54	0.02	0	1.19	0.90
60	53.5	24	58.1	0.54	0.02	0	0.70	1.30
50	46.6	24	48.9	0.54	0.02	0	0.42	1.40
40	38	24	39.4	0.54	0.02	0	0.26	1.90
72	62	24	66.4	0.54	0.02	50	1.09	0.85
71	62	24	65.4	0.54	0.02	100	0.96	0.75
71	62	24	64.8	0.54	0.02	120	0.92	0.85
70	61	24	65.4	0.54	0.02	140	0.91	1.45
70	60.2	24	65.5	0.78	0.02	0	1.36	1.34
70	61.4	24	65.1	0.54	0.02	0	1.07	1.28
70	62.2	24	66.3	0.41	0.02	0	0.96	1.28
70	63.3	24	67.8	0.35	0.02	0	0.88	1.28
70	65.5	24	68.3	0.22	0.02	0	0.64	1.28
70	66.8	24	69.9	0.13	0.02	0	0.44	1.28
50	46.6	24	48.0	0.58	0.02	0	0.37	2.11
50	48.1	24	49.7	0.35	0.02	0	0.28	2.11
50	49.8	24	50.3	0.13	0.02	0	0.11	2.11
70	52.0	24	62.6	0.54	0.008	0	0.91	1.35
60	46.6	24	55.6	0.54	0.008	0	0.64	1.30
51	41.8	24	48.0	0.54	0.008	0	0.36	1.95
41	35.7	24	39.3	0.54	0.008	0	0.23	0.75

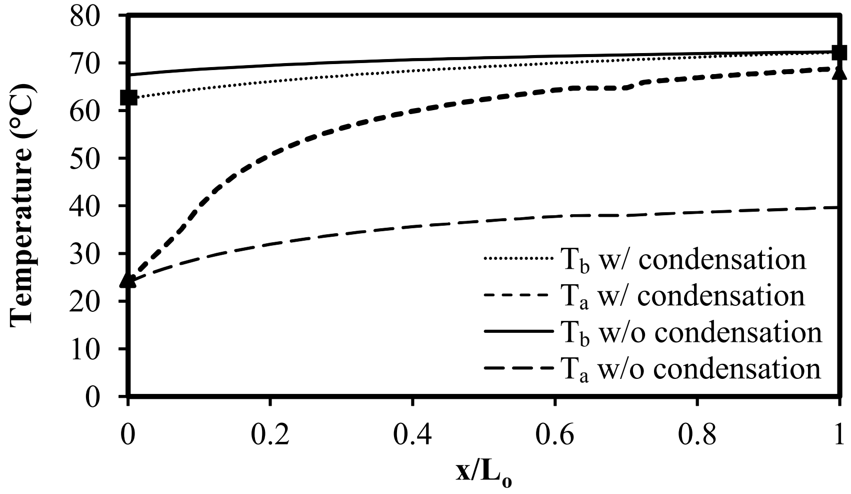

Figure S1. Comparison of predicted brine (T_b) and sweeping gas (T_a) temperature profiles along the length of the module, with and without recondensation for u_a = 0.54 m/s, u_b= 0.02 m/s and brine inlet temperature of T_b = 70 °C. x = 0 at the top of the module (air inlet and brine outlet), and x = L_o at the end of the module (air outlet and brine inlet). The data are provided as symbols.
